# Gas Sensor Based on Surface Enhanced Raman Scattering

**DOI:** 10.3390/ma14020388

**Published:** 2021-01-14

**Authors:** Xu-Ming Wang, Xin Li, Wei-Hua Liu, Chuan-Yu Han, Xiao-Li Wang

**Affiliations:** 1 Department of Microelectronics, Xi’an Jiaotong University, Xi’an 710049, China; xmwang_zw@126.com (X.-M.W.); lwhua@mail.xjtu.edu.cn (W.-H.L.); hanchuanyu@mail.xjtu.edu.cn (C.-Y.H.); 2 School of Physics, Xi’an Jiaotong University, Xi’an 710049, China; xlwang@mail.xjtu.edu.cn

**Keywords:** surface enhanced Raman scattering, ethanol vapor, FDTD simulation, PVP modification, silver nanoparticles

## Abstract

In order to address problems of safety and identification in gas detection, an optical detection method based on surface enhanced Raman scattering (SERS) was studied to detect ethanol vapor. A SERS device of silver nanoparticles modified polyvinylpyrrolidone (PVP) was realized by freeze-drying method. This SERS device was placed in a micro transparent cavity in order to inject ethanol vapor of 4% and obtain Raman signals by confocal Raman spectrometer. We compared different types of SERS devices and found that the modification of polyvinylpyrrolidone improves adsorption of ethanol molecules on surfaces of silver nanoparticle, and finally we provide the mechanism by theory and experiment. Finite Difference Time Domain(FDTD) simulation shows that single layer close-packed Ag nanoparticles have strong local electric field in a wide spectral range. In this study, we provide a case for safety and fingerprint recognition of ethanol vapor at room temperature and atmospheric pressure.

## 1. Introduction

Compared with developed technologies [1] to detect gas molecules such as metal-oxide semiconductor sensor [2,3,4,5], infrared sensor [6,7], photoionization detector [8], electrochemical gas sensor [9,10] and gas chromatograph [11], Raman scattering technology has greater potential and a unique advantage. On the one hand, Raman scattering spectrum shows fingerprint characteristics of gas molecules, which can provide unique identification to detect gas in theory [12,13]. On the other hand, as an optical method, Raman scattering spectrum shows higher safety, which is very suitable for testing combustible gases such as volatile organic compounds (VOCs), oil, and nature gas [14].

In recent years, the rapid development of portable Raman spectrometer has provided more convenience to recognize unknown powders [12,15]. However, compared with solid or liquid, the Raman scattering signal of gas molecule is too weak for its low molecular density and small scattering cross section [16]. Therefore, detection of gases by Raman scattering is still a developing method. Fortunately, surface-enhanced Raman scattering (SERS) technique, which has been widely studied and applied in the realm of biochemistry and medicine, provides another potential approach to detect gas molecules. With the local plasma resonance of noble metal nanoparticles, the Raman scattering light intensity of molecules adsorbed on the surface of nanoparticles could be greatly enhanced up to 10^6^ times [17,18].

However, as for the SERS method, only when molecules lie within a few nanometers of the surface of silver nanoparticle (Ag NPs) could Raman signal be significantly enhanced. Some certain molecules (such as Rhodamine 6G and crystal violet) present a very low limit of detection by SERS method for its special functional group [19], which could be adsorbed on the surface of noble metal particles. For some VOCs molecules like ethanol, we found that adsorption of Ag is too weak to enhance the Raman signal of ethanol directly. It is a good strategy to enrich gas molecules by fabricating metal organic framework (MOF) shells [20] or modifying polymer and organic matter with certain functional groups on nanoparticles, typical organics such as PDMS [21], 4-aminothiophenol (4-ATP), and thiol [22].

In this paper, we show a SERS method to detect ethanol vapor. Here, we give the fabrication process to obtain the SERS device of Ag NPs modified by polyvinylpyrrolidone (PVP) molecules, and a micro transparent chamber was made to place the SERS device. An ethanol vapor generation system was made to obtain 4% ethanol vapor and then vapor would have been put into the micro transparent chamber. Experiments show that the micro chamber with a SERS device could be fully compatible with confocal Raman microscopy and used to detect the Raman signal of ethanol vapor conveniently. Furthermore, SEM was used to characterize the device, a physical model was established and an FDTD algorithm to simulate the surface electric field of nanoparticles. FDTD simulation shows that strong enhancement in a wide spectral range and the higher density of hot spots were the advantages of this device. The comparison experiment showed that PVP-modified SERS devices could improve the adsorption of ethanol molecules. By experiment, we estimate that the maximum adsorption capacity of PVP powders to adsorb ethanol vapor is approximately 16 mL/g. At the same time, we also tried a simple and efficient data processing method to reduce the background of PVP to highlight the signal of ethanol.

## 2. Materials and Methods

Many methods to prepare Ag NPs had been reported [23,24], here we used triethanolamine and AgNO_3_ to prepare Ag NPs.AgNO_3_ as Ag source, triethanolamine as reductant and PVP as surfactant. AgNO_3_ solution of 10 mL at 10 mmol^−1^/L and triethanolamine solution of 20 mL at 10 mmol^−1^/L were set up in two beakers, respectively. Triethanolamine solution was dripped into AgNO_3_ solution gradually by titration until all Ag-ions were converted into silver-amino ions. After that, deionized water was added to the solution to make the total solution volume 50 mL, and 0.1 g PVP powders were added to the above solution and strongly stirred for 15 min. Here the molecular weight of PVP ranged from 25,000 to 40,000. It should be noted that the growth rate of Ag NPs is very slow and is significantly affected by temperature. The solution of silver-amino ions, prepared initially, is clear and transparent. After 48 h, the Ag NPs would gradually grow up and Ag NPs sol could be prepared by this method.

**Figure 1:**
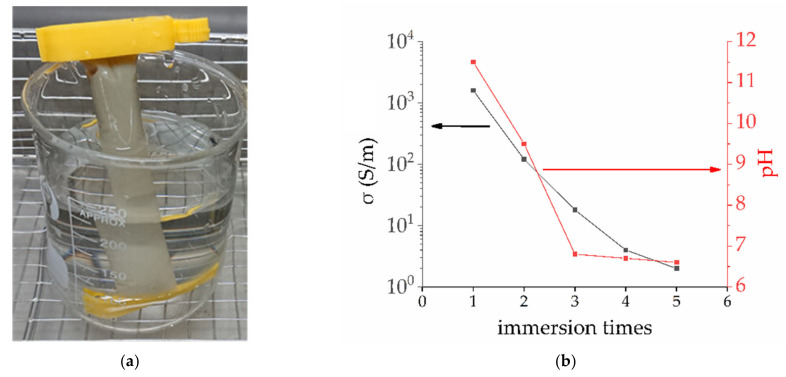
Purification process of Ag sol. (**a**) Dialysis bag filled with Ag sol (gray) was placed in a beaker. (**b**) The conductivity and pH of deionized water decreased with the increase of immersion times.

There was 20 mL Ag NPs sol put into the dialysis bag, and then the dialysis bag was put into the beaker with deionized water. The dialysis bag allows only molecules and ions below a certain diameter to pass through. Here, the dialysis bag we used is a commercial specification of MW3500(average relative molecular weight less than 3500), which could keep PVP and Ag NPs. When the dialysis bag was soaked in a beaker full of deionized water, the ammonium, nitrate ions and other small molecules in the bag will have been diluted by deionized water. By testing the deionized water in beaker with conductivity tester and pH meter, we can know the quality of Ag NPs sol indirectly. This process was shown in Figure 1. The pH and conductivity of deionized water were measured and recorded after immersion for 2 h. In order to obtain Ag NPs sol with very low ion concentration, dialysis bag with Ag NPs must have been soaked for many times by deionized water. Figure 1b shows that the pH and conductivity of deionized water gradually decreases during the 5 soaking processes. In general, when the conductivity was less than 0.1, this purification process would be over.

**Figure 2:**
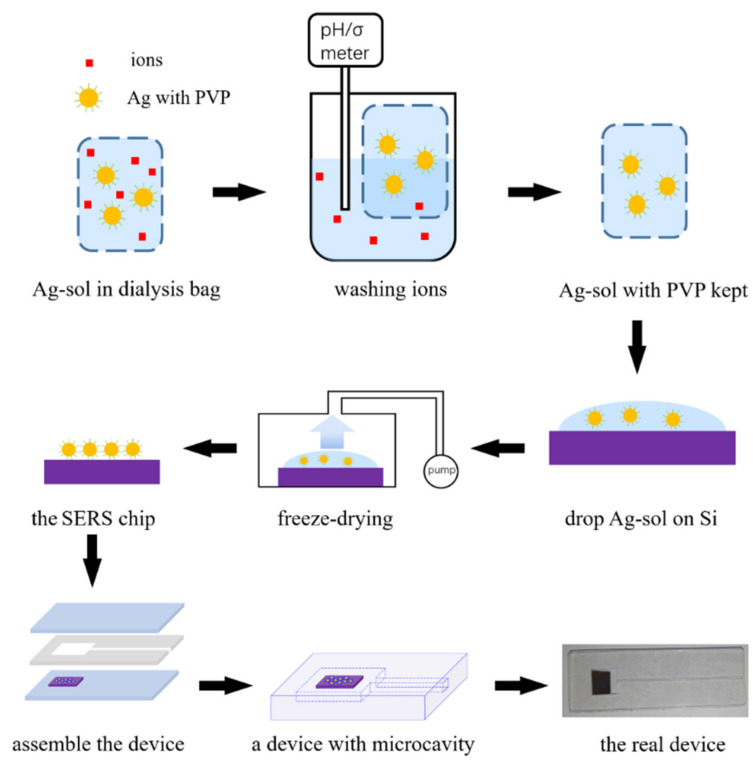
Fabrication process of the surface-enhanced Raman scattering (SERS) device for ethanol testing.

There was 20 μL of Ag NPs sol dripped on 5 mm × 5 mm Si substrate with a pipette. The substrate was put into the freeze-drying machine at −15 °C and 0.1 Pa for 12 h, and then a SERS device was made. The SERS device was put into a micro transparent chamber that made of glass and silicone rubber. By this method, we can fabricate miniature glass chambers easily, which are highly compatible with the confocal Raman spectrometer. Figure 2 shows the entire process to fabricate a device.

**Figure 3:**
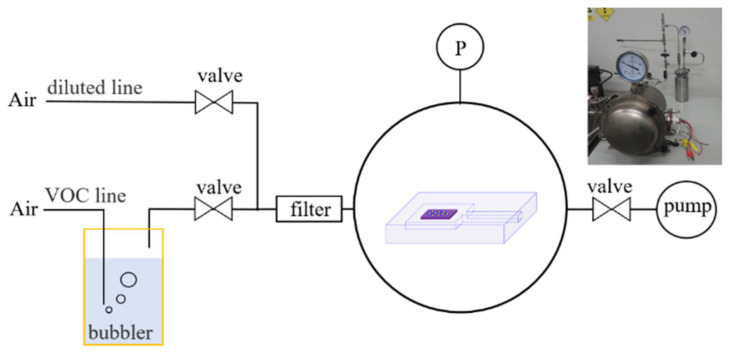
Schematic diagram of ethanol production and gas delivery system. On the top right is a picture of the real system.

The VOC vapor generation system includes a bubbler to generate ethanol vapor, a filter to remove dust from the air, a big vacuum chamber to place the transparent glass chamber, a pump, a pressure gauge and several valves. Figure 3 shows the schematic diagram and a real picture of vapor system.

**Figure 4:**
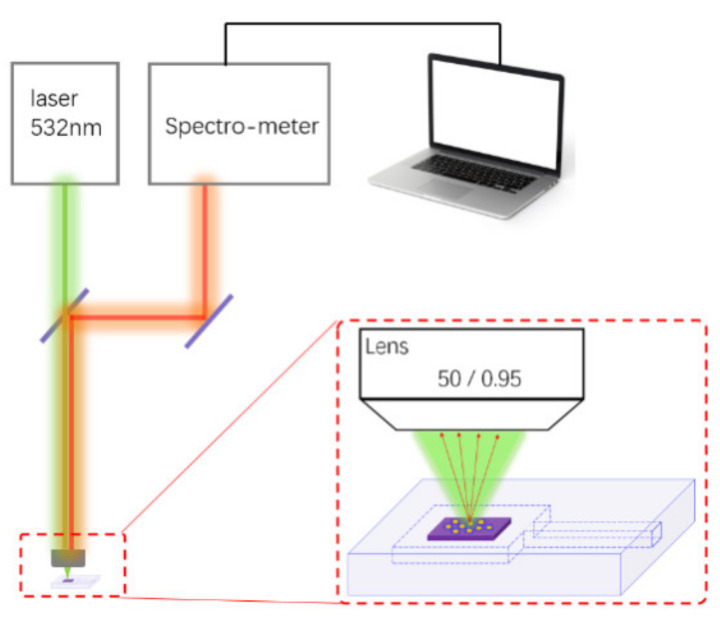
Schematic diagram of ethanol sensor with a SERS chip Raman spectrometer test.

According to Antoine’s equation, the saturated vapor pressure of ethanol could be expressed as lg P = 8.2133 − 1652/(T + 231.48). According to this equation, a maximum concentration of 8% ethanol vapor can be obtained by controlling the temperature (25 °C) of the ethanol in the bubbler. By changing the gas ratio between the two lines (ethanol vapor from VOC line and air from diluted line), we could control the concentration of ethanol vapor. The micro transparent chamber with a SERS device was placed in the big chamber, and then the big chamber was vacuumed. After this process, ethanol vapor of 4% was injected into the big chamber and the micro transparent chamber as well. Finally, the micro transparent chamber with 4% ethanol vapor could be placed in a confocal Raman spectrometer to test. Here, we use a confocal Raman spectrometer (HORIBA LabRAM HR-800, HORIBA JOBIN YVON S.A.S, Paris, France), the incident light wavelength is 532 nm, and the laser power irradiated to the sample is 1 mw. The cold Charge Coupled Device (CCD) as a photon detector in this spectrometer and exposure time of it is 60 s. Here, the schematic diagram was shown in Figure 4.

## 3. Results

### 3.1. The Spectrum Test and Calibration of Ethanol Vapor

**Figure 5:**
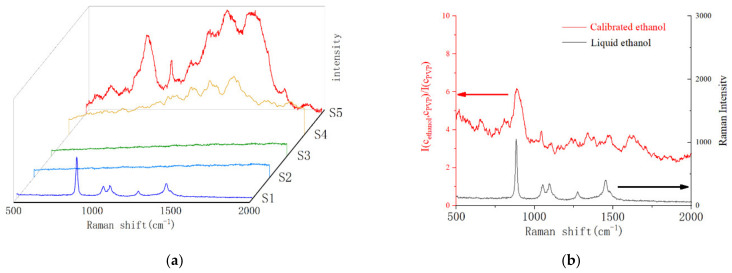
(**a**) Raman spectra of 5 samples. (**b**) Comparison of Raman spectra of liquid ethanol and gas ethanol, our algorithm reduces the interference peak of PVP.

In order to compare the enhancement effect of Ag NPs on different substances, 5 samples were prepared, respectively, S1 is the liquid ethanol in glass bottles, S2 is the SERS device filled with air in the chamber, S3 is the SERS device filled with 4% ethanol in the chamber, and Ag NPs of S2 and S3 were not modified PVP molecules. S4 was the SERS device filled with air, Ag NPs was modified by PVP.S5 was a SERS device filled with 4% ethanol and Ag NPs was modified by PVP. The Raman spectra of five samples shown in Figure 5a. Standard spectra of ethanol molecules obtained from S1 samples. It can be seen from S2 and S3 that the unmodified SERS devices cannot enhance the air or ethanol vapor. These two samples also confirmed that the enhancement of Ag NPs was a short-range action, and adsorption of molecules on the surface of Ag NPs was an important and prerequisite condition for SERS. The Raman spectra of PVP and ethanol molecules were observed in S4 and S5 samples. Due to the presence of PVP molecules modified on Ag NPs, additional interferential peaks ranging from 1200 to 2000 were introduced into the SERS device. In order to eliminate the interferential peaks as much as possible, we here try a simple algorithm to calibrate spectrum of S5 by S4. The Raman spectra processed by our algorithm were shown in Figure 5b.

The whole Raman spectrum can be represented as I*_λ_*, here subscript *λ* for Raman shift and ranges from 500 to 2000. The spectral of Ag NPs with PVP could be expressed as $I_{\lambda }^{\mathrm{standard}}\left(C_{\mathrm{PVP}}\right)$ (the S4 of Figure 5a). After ethanol molecules captured by PVP, Raman spectral intensity was expressed as I*_λ_*(*C*_ethanol_, *C*_PVP_) (the S5 of Figure 5a).

(1) $$I_{\lambda }\left(C_{\mathrm{ethanol}},C_{\mathrm{PVP}}\right)=I_{\lambda }\left(C_{\mathrm{ethanol}}\right)+{\;I}_{\lambda }\left(C_{\mathrm{PVP}}\right)+\;\alpha {\left[I_{\lambda }\left(C_{\mathrm{ethanol}}\right){*I}_{\lambda }\left(C_{\mathrm{PVP}}\right)\right]}^{\frac{1}{2}}$$

Here, $\alpha {\left[I_{\lambda }\left(C_{\mathrm{ethanol}}\right){*I}_{\lambda }\left(C_{\mathrm{PVP}}\right)\right]}^{\frac{1}{2}}$ represents the interaction from two kinds of molecules, α is an undetermined coefficient, and power of 1/2 make sure the dimension of whole formula is uniform.$\frac{I_{\lambda }\left(C_{\mathrm{ethanol}},C_{\mathrm{PVP}}\right)}{I_{\lambda }^{\mathrm{standard}}\left(C_{\mathrm{PVP}}\right)}$ could be expressed as:

(2) $$\frac{I_{\lambda }\left(C_{\mathrm{ethanol}},C_{\mathrm{PVP}}\right)}{I_{\lambda }^{\mathrm{standard}}\left(C_{\mathrm{PVP}}\;\right)}=\frac{I_{\lambda }\left(C_{\mathrm{ethanol}}\right)}{I_{\lambda }^{\mathrm{standard}}\left(C_{\mathrm{PVP}}\right)}+\frac{I_{\lambda }\left(C_{\mathrm{PVP}}\right)}{I_{\lambda }^{\mathrm{standard}}\left(C_{\mathrm{PVP}}\right)}+\frac{\alpha {\left[I_{\lambda }\left(C_{\mathrm{ethanol}}\right)*I_{\lambda }\left(C_{\mathrm{PVP}}\right)\right]}^{\frac{1}{2}}}{I_{\lambda }^{\mathrm{standard}}\left(C_{\mathrm{PVP}}\right)}$$

Here the Equation (3) could exist

(3) $$I_{\lambda }^{\mathrm{standard}}\left(C_{\mathrm{PVP}}\right)\approx {k\;I}_{\lambda }\left(C_{\mathrm{PVP}}\right)$$

so, Equation (2) could be written as follows

(4) $$\frac{I_{\lambda }\left(C_{\mathrm{ethanol}},C_{\mathrm{PVP}}\right)}{I_{\lambda }\left(C_{\mathrm{PVP}}\right)}=\;\frac{I_{\lambda }\left(C_{\mathrm{ethanol}}\right)}{I_{\lambda }\left(C_{\mathrm{PVP}}\right)}\;+\;\alpha {\left[\frac{I_{\lambda }\left(C_{\mathrm{ethanol}}\right)}{I_{\lambda }\left(C_{\mathrm{PVP}}\right)}\right]}^{\frac{1}{2}}+k$$

The interference of PVP can be deducted by $\frac{I_{\lambda }\left(C_{\mathrm{ethanol}},C_{\mathrm{PVP}}\right)}{I_{\lambda }\left(C_{\mathrm{PVP}}\right)}$, and a calibrated spectrum of ethanol (the red spectrum of Figure 5b) can be obtained. From the red spectrum in Figure 5b, we can find that the background of PVP is inhibited to a low degree from 1000 to 2000, while the peak of ethanol is relatively obvious. The spectrum of S1 was attached in Figure 5b for convenience of comparison. By comparing the spectra of liquid and gas ethanol, we can find the peaks of ethanol on Ag NPs modified by PVP.

### 3.2. The Microstructure and Simulation of SERS Device

**Figure 6:**
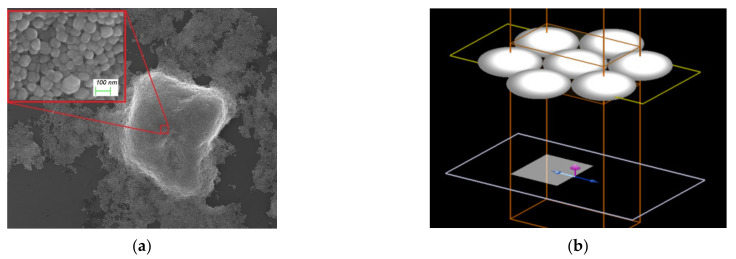
Microstructure of Ag NPs of SERS device. (**a**) Ag NPs on Si observed under scanning electron microscope, and (**b**) Model for FDTD simulation of single layer hexagonal stacked Ag particles.

The stacking form of Ag NPs in SERS device was been analyzed by microscope (at Figure 6). SEM shows that Ag NPs on Si prepared by freeze-drying tend to be packed in dense stacks. The diameter of a single nanoparticle is about 60 nm, pores formed between Ag NPs. Considering the tradeoff between real situations and computational load, we believe that this form of dense packing could be described approximately by the single layer hexagonal close-packed (SL-HCP) model.

It is important to note that the two-step method is the standard method for synthesizing Ag NPs with a diameter of 60 nm in many reports, but we provide a method to obtain Ag NPs with a diameter of 60 nm directly in our study. Our analysis suggests that reduction rate of AgNO_3_ by ethanolamine is very slow, which makes Ag NPs have sufficient time to further grow up after the nucleation process.

**Figure 7:**
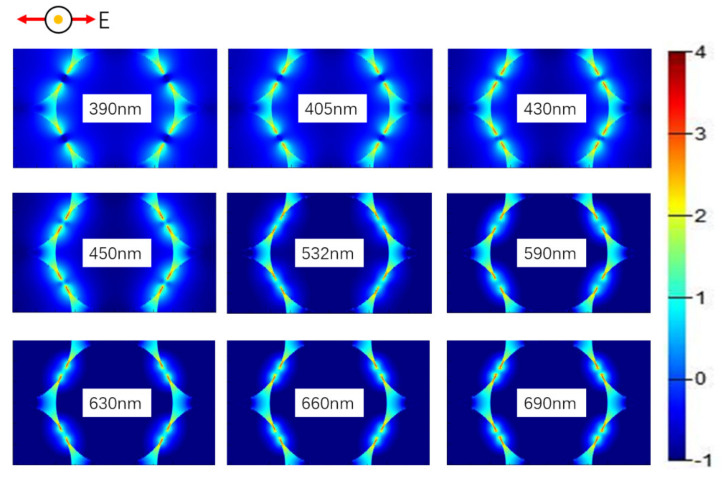
Electric field distribution of Ag NPs stacked by SL-HCP. The color bar on the far right represents an order of magnitude of E^2^. The red arrow in the upper left corner is the direction of the electric field vibration of the plane wave, and the black circle of the yellow dot is the direction of the energy flow.

**Figure 8:**
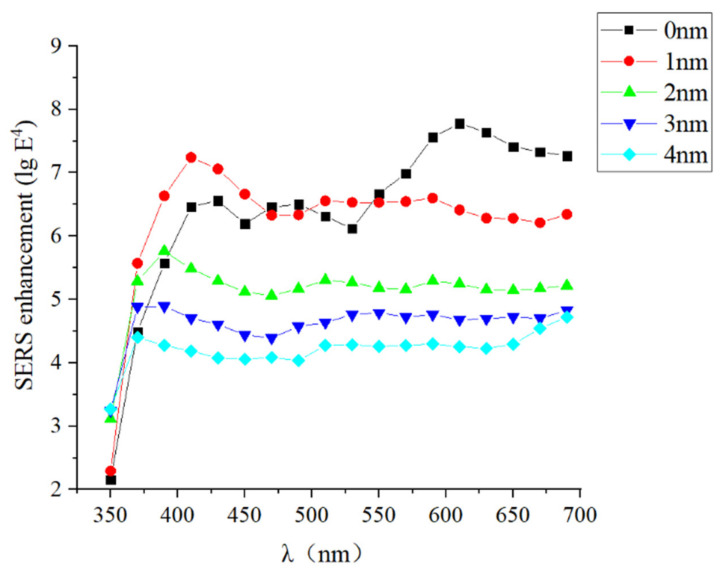
Order of E^4^ at different gap and wavelength. Total enhancement factor of SERS is proportional to E^4^, when the gap is 0 nm and wavelength is 630 nm, total enhancement factor increased by about 8 orders of magnitude.

In report [25], the author has studied the local electric field of some ideal models, so our study pays more attention to practical situation. Compared with the enhancement of a two-sphere SERS system, SL-HCP stacking can obviously obtain a higher density of hot spots, and process to fabricate is easier as well. Following this logic, FDTD algorithm was used to stimulate the electric field distribution of Ag NPs. Simulation parameters were as follows, periodic boundary conditions of SL-HCP structure are adopted, the minimum mesh size is 0.1 nm at the gap, the incident light is plane wave and wavelength of it ranges from 350 nm to 700 nm, and here the diameter of Ag sphere is 60 nm. It was found that Ag NPs tend to form extremely strong localized electric fields under the SL-HCP structure as well, and local electric field intensity decreases with the increase of gap. E^2^ and E^4^ are the key parameters we were concerned about. It is generally believed that single-step enhancement factor is proportional to E^2^ and the total enhancement factor is proportional to E^4^ [25]. The E^2^ mapping of SL-HCP structure was shown in Figure 7. The order of magnitude of E^4^ under different gaps and wavelengths was shown in Figure 8, and we can conclude that SERS-enhanced factors increased by approximately 6 to 8 orders of magnitude as well when SL-HCP structure was adopted.

By observing Figure 7, we know that one Ag NP could produce 4 hot spots under excitation of plane wave polarized light. The diameter of one Ag NP is 6 × 10^−6^ cm, so we can estimate the number of hot spots is about 6.4 × 10^10^/cm^2^. Figure 7 shows that SL-HCP structure has a high enhancement effect in the visible light range. Obviously, we can draw a conclusion that the total Raman enhancement factor can affect the SNR of Raman spectrum, whereas the density of hot spots and laser power can affect the spectral intensity. The density of hot spots and the total enhancement factor determine the detection limit and identification ability of the SERS device.

In the actual situation, it is necessary to use the Laser Particle Analyzer to obtain the distribution percentage of Ag NPs number and diameter, as well as the hot spot density and total enhancement factor at different diameters, so as to calculate the average enhancement effect per unit area.

## 4. Discussion

### 4.1. Mechanism and Capacity of Adsorption about PVP on SERS Device

We know that under standard condition every 22.4 L of air contains 1 mole of molecules of gas (oxygen, nitrogen and CO_2_), that is, one molecule (N_2_, O_2_ or CO_2_) can be found per 37 nm^3^ volume. The diameter of the Ag sphere is 60 nm, and in the single-layer SL-HCP structure, it can be calculated that approximately 39.5% of the space can accommodate gas molecules, that is, porosity is about 39.5%. The triangular structure consists of three spheres, with a space of about 37,000 nm^3^, which can hold about 1000 gas molecules. Because the actual nanoparticles are not ideal spheres, and the particle diameter is distributed within a certain range, the porosity will be less than 39.5%. Even so, within the laser spot (about 1 micron in diameter) there should be a large number of enhanced gas molecules. However, the test results of S2 and S3 samples showed that this was not the case. If the short-range action of hot spot is considered, for every 37,000 nm^3^, the volume of the E^4^ exceeding 10^6^ is about 5 nm^3^, which accounts for only about 0.014% of the total space. From this calculation and analysis, we can see that about 0.1 gas molecules can be found in the volume occupied by each hot spot on average. This explains why SERS could not enhance the Raman light of atmosphere and ethanol. On the other hand, considering that molecules (nitrogen, oxygen, CO_2_) still have some kinetic energy after being adsorbed on the surface of Ag and migrate on the surface, gas molecules are not confined to the hot spot.

**Figure 9:**
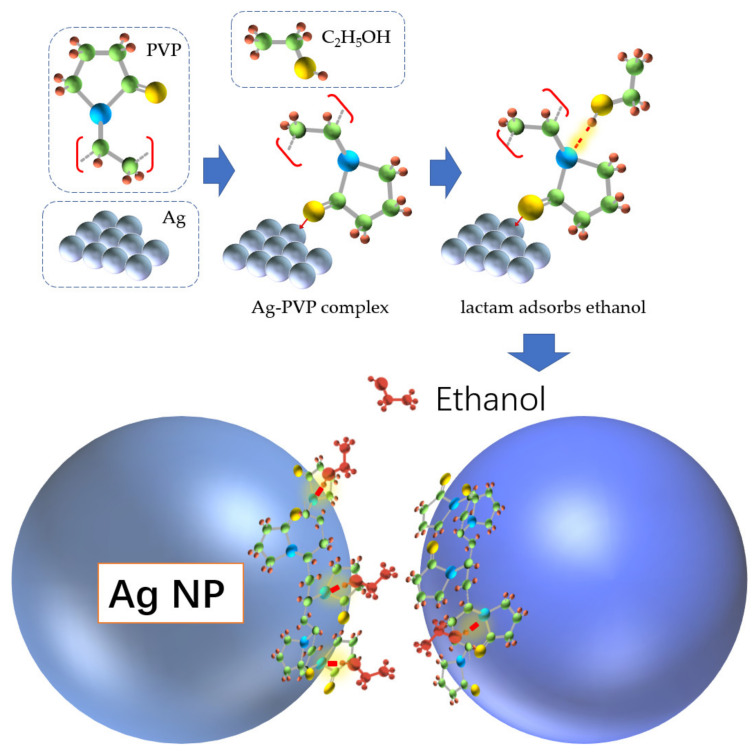
In the preparation of Ag NPs, Ag atoms grew into Ag NPs on PVP templates [28]. After freeze-drying, PVP molecules were retained and attached to Ag NPs. Ethanol molecules are trapped on the surface of Ag NPs by hydrogen bonding from the lactam group of PVP. When the PVP located in the gap between the two Ag NPs captures the ethanol, the Raman light intensity is enhanced by local electric field.

Due to the special molecular structure of PVP, the carbonyl functional group can complexate with Ag atoms and adhere to Ag NPs stably [26]. On the other hand, the presence of polar lactam groups can capture polar molecules by hydrogen bonding [27]. Figure 9 shows the mechanism of this bridging action. This capture enables the ethanol to be close to the Ag NPs surface and to enhance its Raman intensity by means of the local electric field.

In synthesis process, the total amount of Ag NO_3_ is 1 × 10^−4^ mol, and the total amount of PVP (molecular weight 25,000~40,000, average molecular weight 38,000) is about 2.6 × 10^−6^ mol. When the diameter of Ag NPs was 60 nm, we estimated that each Ag NP wound about 170,000 PVP molecular chains on average. There are about 5.8 × 10^7^ monomers of vinyl pyrrolidone on the surface of each Ag NP. The surface area of Ag spheres with a diameter of 60 nm is about 11,304 nm^2^, so the density of monomers on the surface of Ag spheres is about 15/nm^2^. It is noteworthy that this calculation is the density of the Ag NPs sol. In the freeze-drying process, PVP molecules may undergo chemical segregation due to the cooling process, and the actual density of NVP monomers on the Ag NPs surface should be less than the calculated value. In the process of making SERS devices, it was a difficult problem to keep pure PVP molecular chains on the surface of Ag NPs. Therefore, we used the dialysis method and freeze-drying method to make SERS devices. The purpose is to use the functional groups of PVP molecules to adsorb ethanol.

**Figure 10:**
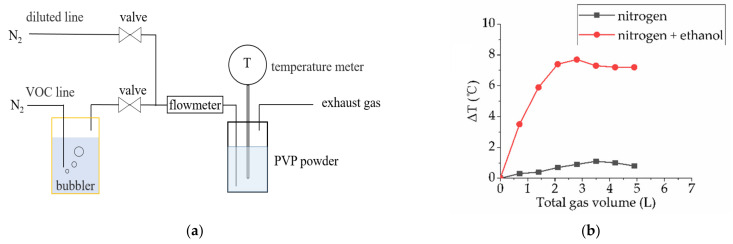
(**a**) The ethanol vapor was injected into the container with 10 g PVP powders. (**b**) The temperature difference (ΔT) of PVP powders was tested with a thermometer.

In this study, to evaluate the adsorption capacity of PVP on ethanol, we designed a simple and effective method to observe the adsorption of ethanol molecules onto PVP. Based on our equipment, 10 g PVP powders was put in a vessel, and a small digital thermometer was used to test the temperature of the powders. As shown in Figure 10a, pure N_2_ was transported in the bubbler as the carrier gas to obtain 8% ethanol vapor. When ethanol vapor was injected into the vessel, temperature of powders started to heat up in time. The temperature difference could be calculated from process temperature and initial temperature. The temperature rise of PVP indicates that the process of ethanol adsorption is a physical and exothermic process, which is completely consistent with our expectation.

As shown in Figure 10b, the temperature difference of red curve rises to a certain degree (about 8 °C) and starts to flatten after 2 L of mixture gas was injected in. This process indicates that PVP powders start to saturate. By this method, we can roughly estimate that the maximum adsorption capacity of PVP powders to adsorb ethanol vapor is approximately 16 mL/g. N_2_ as a medium was also tested to deduct the throttling process of vessel and adsorption of N_2_, and we can be sure that the adsorption of N_2_ by PVP is very weak from the black curve in Figure 10b. In addition, similar exothermic tests have been performed on a variety of substances (polyethylene glycol powder and molecular sieve powder). Considering the process simplicity, PVP is the best modifier for Ag NPs.

### 4.2. Enhancement Effect of Non-Surface Ag NPs on SERS

**Figure 11:**
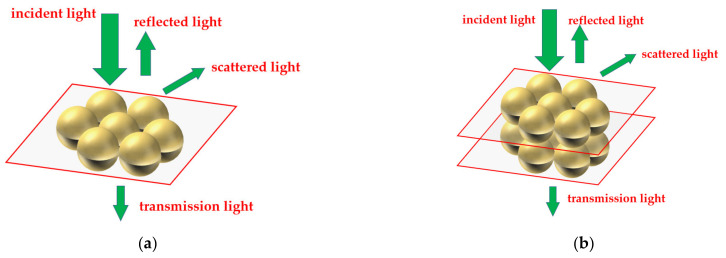
Interactions of Ag NPs with incident light and their geometry. (**a**) single-layer Ag NPs and (**b**) double-layer Ag NPs.

In the SEM images (Figure 6a), we noticed that there were a large number of non-surface Ag NPs in the SERS device. In this study, the enhancement of Ag NPs of the second layer (under the surface layer of Ag NPs) was calculated by FDTD simulation. In the SL-HCP structure of monolayer Ag NPs, the incident light is partly transmitted, partly reflected, and the rest scattered (Figure 11a). In the real device, part of the transmitted light should be able to further stimulate the second layer Ag NPs to generate electric field enhancement (Figure 11b). The double layer HCP structure was used to approximate this effect.

**Figure 12:**
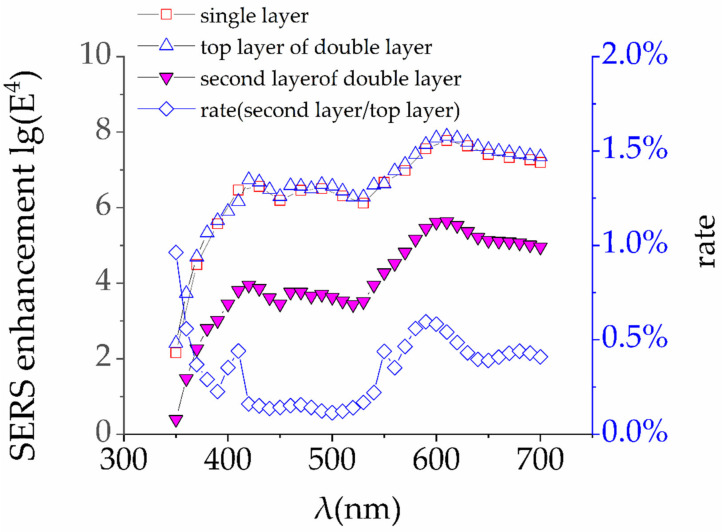
We compare the E^4^ obtained by the top layer of the double layer and the single layer, and find that two curves are almost identical. The second layer only achieves an enhancement of 4 to 6 orders of magnitude. Compared with the top layer, the enhancement of the second layer is only about 0.1 to 0.6%.

Here, we still take the single-layer model with a gap of 0 nm as a comparison, and calculate the maximum of E^4^ of the top layer and the second layer (Figure 12). The ratio of E^4^ of the second layer to the top layer was shown as well.

**Figure 13:**
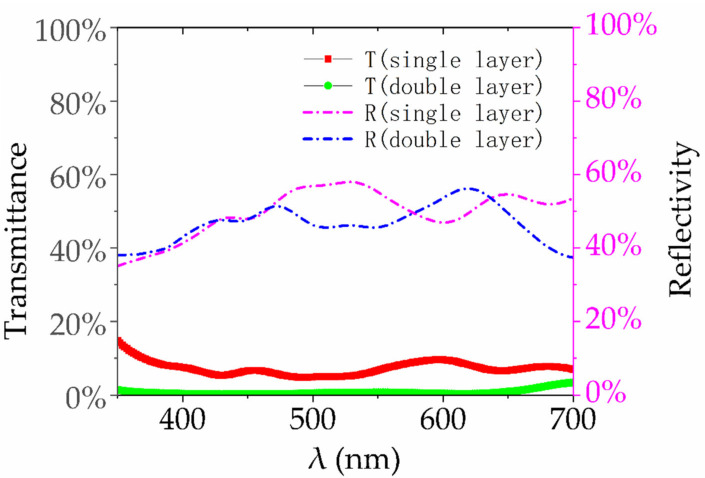
Transmittance and reflectance curves of Ag NPs for single layer and double layer. Reflectivity and transmittance show that most of the light is reflected and scattered by the first layer. The transmittance of single layer is about 10%, and that of double layer is about 1%.

Furthermore, the reflectivity and transmittance of Ag NPs are calculated (Figure 13). Simulation results show that for a single layer of Ag NPs, most of the incident light is reflected and scattered (the scattered light can be calculated as 1-T-R), and only a small part (about 10%) can penetrate the top layer to the second layer.

We believe that factors of transmittance and reflectivity limit the further enhancement of Ag NPs. In our opinion, the transmittance and reflectivity curves might mean that the hollow shell of Ag NPs or Ag shell with SiO_2_ nucleus or the resonator/reflection cavity with reasonable geometric structure could further improve the enhancement effect.

## 5. Conclusions

In this study, we prepared SERS devices using Ag NPs and obtained the Raman spectrum of ethanol vapor. In order to improve the ability of SERS devices to capture ethanol molecules, we used PVP as the adsorption medium. The surface electric field distribution of Ag NPs was been studied by FDTD simulation. Finally, we measured the adsorption and exothermic process of PVP to evaluate the adsorption capacity of PVP to ethanol. The overall results showed that SERS method to detect VOCs like ethanol had better potential.

## Data Availability

The data presented in this study are available on request from cor-responding author.

## References

[1] Spinelle L., Gerboles M., Kok G.J.P., Persijn S., Sauerwald T. (2017). Review of Portable and Low-Cost Sensors for the Ambient Air Monitoring of Benzene and Other Volatile Organic Compounds. Sensors.

[2] Walker J.M., Akbar S.A., Morris P.A. (2019). Synergistic effects in gas sensing semiconducting oxide nano-heterostructures: A review. Sens. Actuators B Chem..

[3] Nazemi H., Joseph A., Park J., Emadi A. (2019). Advanced Micro- and Nano-Gas Sensor Technology: A Review. Sensors.

[4] Yuan Z., Li R., Meng F., Zhang J., Zuo K., Han E. (2019). Approaches to Enhancing Gas Sensing Properties: A Review. Sensors.

[5] Moseley P.T. (2017). Progress in the development of semiconducting metal oxide gas sensors: A review. Meas. Sci. Technol..

[6] Hodgkinson J., Tatam R.P. (2012). Optical gas sensing: A review. Meas. Sci. Technol..

[7] Bogue R. (2015). Detecting gases with light: A review of optical gas sensor technologies. Sens. Rev..

[8] Rezende G.C., Le Calvé S., Brandner J.J., Newport D. (2019). Micro photoionization detectors. Sens. Actuators B Chem..

[9] Tong P., Liang J., Jiang X., Li J. (2019). Research Progress on Metal-Organic Framework Composites in Chemical Sensors. Crit. Rev. Anal. Chem..

[10] Baron R., Saffell J. (2017). Amperometric Gas Sensors as a Low Cost Emerging Technology Platform for Air Quality Monitoring Applications: A Review. ACS Sens..

[11] Bhardwaj S.K. (2016). A Review: GC Method Development and validation. Int. J. Anal. Bioanal. Chem..

[12] Jehlička J., Vitek P., Edwards H.G., Hargreaves M.D., Čapoun T. (2009). Fast detection of sulphate minerals (gypsum, anglesite, baryte) by a portable Raman spectrometer. J. Raman Spectrosc..

[13] Muehlethaler C., Leona M., Lombardi J.R. (2016). Review of Surface Enhanced Raman Scattering Applications in Forensic Science. Anal. Chem..

[14] Sharma R., Poonacha S., Bekal A., Vartak S., Weling A., Tilak V., Mitra C. (2016). Raman analyzer for sensitive natural gas composition analysis. Opt. Eng..

[15] Košek F., Culka A., Rousaki A., Vandenabeele P., Jehlička J. (2020). Evaluation of handheld and portable Raman spectrometers with different laser excitation wavelengths for the detection and characterization of organic minerals. Spectrochim. Acta Part A Mol. Biomol. Spectrosc..

[16] Fenner W.R., Hyatt H.A., Kellam J.M., Porto S.P.S. (1973). Raman cross section of some simple gases. J. Opt. Soc. Am..

[17] Nie S., Steven R. (1997). Emory, Probing Single Molecules and Single Nanoparticles by Surface-Enhanced Raman Scattering. Science.

[18] Kneipp K., Wang Y., Kneipp H., Perelman L.T., Itzkan I., Dasari R.R., Feld M.S. (1997). Single Molecule Detection Using Surface-Enhanced Raman Scattering (SERS). Phys. Rev. Lett..

[19] Yan W., Yang L., Chen J., Wu Y., Wang P., Li Z. (2017). In Situ Two-Step Photoreduced SERS Materials for On-Chip Single-Molecule Spectroscopy with High Reproducibility. Adv. Mater..

[20] Qiao X., Su B., Liu C., Song Q., Luo D., Mo G., Wang T. (2018). Selective Surface Enhanced Raman Scattering for Quantitative Detection of Lung Cancer Biomarkers in Superparticle@MOF Structure. Adv. Mater..

[21] Qian C., Guo Q., Xu M., Yuan Y., Yao J. (2015). Improving the SERS detection sensitivity of aromatic molecules by a PDMS-coated Au nanoparticle monolayer film. RSC Adv..

[22] Xia D., Guo Q., Ge M., Yuan Y., Xu M., Yao J. (2016). On-line sensitive detection of aromatic vapor through PDMS/C3H7S-assisted SERS amplification. RSC Adv..

[23] Kaushik M., Moores A. (2016). Review: Nanocelluloses as versatile supports for metal nanoparticles and their applications in catalysis. Green Chem..

[24] Barud H.S., Regiani T., Marques R.F.C., Lustri W.R., Messaddeq Y., Ribeiro S.J.L. (2011). Antimicrobial Bacterial Cellulose-Silver Nanoparticles Composite Membranes. J. Nanomater..

[25] Ding S.-Y., Yi J., Li J.-F., Ren B., Wu D.-Y., Panneerselvam R., Tian Z.-Q. (2016). Nanostructure-based plasmon-enhanced Raman spectroscopy for surface analysis of materials. Nat. Rev. Mater..

[26] Wang H., Qiao X., Chen J., Wang X., Ding S. (2005). Mechanisms of PVP in the preparation of silver nanoparticles. Mater. Chem. Phys..

[27] Saidi W.A., Feng H., Fichthorn K.A. (2013). Binding of Polyvinylpyrrolidone to Ag Surfaces: Insight into a Structure-Directing Agent from Dispersion-Corrected Density Functional Theory. J. Phys. Chem. C.

[28] Koczkur K.M., Mourdikoudis S., Polavarapu L., Skrabalak S.E. (2015). Polyvinylpyrrolidone (PVP) in nanoparticle synthesis. Dalton Trans..

